# Chemical Resistance of Modified Wood Veneers in Sustainable Load Bearing Elements

**DOI:** 10.1021/acsomega.4c07320

**Published:** 2024-11-20

**Authors:** Sebastian Wurm, Alexa Scheer, Georg Baumann, Markus Wagner, Kevin Vitzthum, Stefan Spirk, Florian Feist

**Affiliations:** † Vehicle Safety Institute, Graz University of Technology, Inffeldgasse 13/6, 8010 Graz, Austria; ‡ Institute of Bioproducts and Paper Technology, Graz University of Technology, Inffeldgasse 23, 8010 Graz, Austria; § W.E.I.Z. Forschungs & Entwicklungs gGmbH, Franz-Pichler-Straße 30, 8160 Weiz, Austria

## Abstract

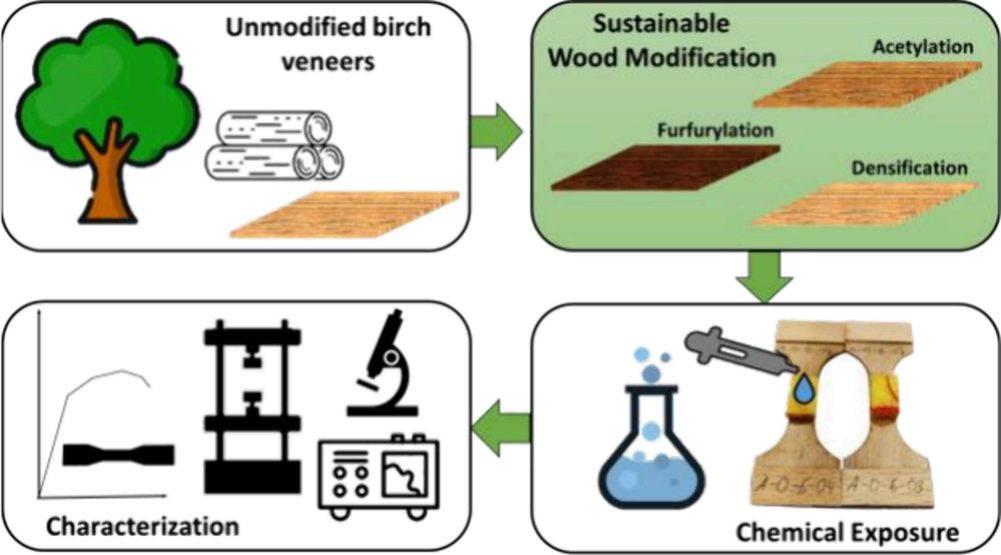

In the pursuit of sustainable engineering solutions, material selection is increasingly directed toward resources that offer functional efficacy, economic feasibility, and minimal environmental impact. To replace environmentally damaging materials like aluminum with more sustainable alternatives like wood-based materials, it is essential to improve the durability and longevity of wood. This study explores the potential suitability of modified veneers as an outer protective layer for unmodified wooden load-bearing elements, providing a cost-effective and resource-efficient alternative to bulk modification. Unmodified, acetylated, furfurylated, and physically densified birch rotary-cut wood veneers were exposed to liquid chemical reagents (acids, base, solvents, and water) and characterized thereafter in tensile tests. The chemical resistance was evaluated based on the deterioration of tensile strength. Additionally, infinite focus microscopy, infrared spectroscopy, and contact angle measurements were performed to track morphological and chemical changes in the veneers. The results demonstrated that acetylation and furfurylation significantly enhanced chemical resistance against the tested reagents.

## Introduction

Aluminum, with its exceptional characteristics such as low density, high strength, and resistance to corrosion, has long held a central role in various industries and subsequent products. One of the most important applications is within the automotive sector, where aluminum has contributed to lightweight design and improved fuel efficiency. However, the ecological footprint left by aluminum through its entire lifecycle—from bauxite mining to processing—raises significant environmental concerns. The substitution of aluminum by more sustainable products would lead to benefits in terms of ecological footprint and CO_2_ equivalents.^1−3^ In this context, wood and wood-based products such as veneers have entered the focus of research.^4−6^ Wood itself is a composite material with a complex ultrastructure providing exceptional load bearing properties for trees. In addition, wood is commonly regarded as a highly resistant material against aggressive substances, which is why it is widely used for construction of industrial buildings that house chemically aggressive agents. For example, within plants dedicated to graphitizing and anodizing, the wooden roof frameworks endure prolonged exposure to hot steam and a combination of sulfuric, chromic, and oxalic acids over decades, yet they demonstrate resilience, showing no risk of structural failure. While wooden constructions are susceptible to deterioration from harsh chemical substances, research indicates a consistent trend contributing to their durability as the damage is mostly limited to the outermost layer (10–15 mm). The mechanical properties of the wood beneath this layer are not significantly impacted compared to undamaged wood.^7^ This circumstance necessitates the upsizing of structural components. While this may not significantly impact the construction industry, it poses a considerable challenge within the realm of mechanical engineering.^8,9^ Evidence indicates that wood demonstrates a robust resilience against environments with moderate acidity or alkalinity, especially within a pH range between 2 to 11. It is primarily the more extreme conditions—strong acids with pH levels below 2 and highly alkaline solutions with pH above 11—that are likely to significantly deteriorate the mechanical characteristics of wood samples.^10,11^

Nevertheless, it is critical to consider that factors beyond pH and concentration also play a decisive role in the corrosion processes affecting wood. A strategy to increase stability is to implement veneers as surface coating to the wood of interest. Here, particularly the use of chemically or mechanically modified veneers,^12^ is a promising strategy to increase the chemical resistance. Especially, acetylation and furfurylation are interesting modification pathways, as these can be performed also via the gas phase, avoiding the use of solvents, thereby improving their environmental footprint. In addition, the relevant starting materials (acetic acid anhydride, furfuryl anhydride) are already produced at industrial scale from biobased feedstock.^13^

### Acetylation

The esterification of hydroxyl groups through a reaction with acetic anhydride results in a lower amount of accessible hydroxyl groups and thus lower hydrophilicity of the wood. Reducing swelling/shrinkage by up to 75%^14^ and increasing durability against microorganisms to the highest class “very durable”.^15^ Furthermore, acetylated wood exhibits great resistance against marine borers such as mollusks and crustaceans on par with chromated copper arsenate (CCA)^16^ as well as subterranean termites.^17^ The influence of acetylation on the mechanical properties is less certain. Some sources conclude that the properties remain unchanged^18,19^ while others point out that this is highly dependent on the wood species^20^ as well as the impregnation time.^21^

### Furfurylation

Furfuryl alcohol (C_5_H_6_O_2_) is commonly sourced from agricultural waste (corn cobs, sugar cane bagasse). Furfurylated wood is produced by using a mixture of furfuryl alcohol and catalysts, which are polymerized through heat treatment. The toxicity of furfuryl alcohol is strongly reduced after polymerization^13^ and studies showed that furfurylated wood exhibits no significant ecotoxicity.^22,23^ The exact mechanism of furfurylation is rather complex and not completely understood at this point, as it is not clear if the furfuryl alcohol reacts only with itself^24^ or polymerizes directly with the wooden cell walls.^25^ Nevertheless, the furfuryl alcohol complexes partly fill the wood cavities as well as the cell walls, thereby increasing hydrophilicity and density at the same time.

### Densification

Many mechanical properties of wood are determined by its density, as higher densities correlate with increased strength and hardness, among other properties. Densification is used to enhance the competitiveness of low-density species by compressing the cell structure and thus improving upon the mechanical properties. Densification, in a general sense is a thermo-hydro-mechanical process and does not involve any additives.^26^ However, one of the challenges of densified wood is the moisture-induced shape recovery. Untreated and not impregnated densified wood will swell back toward the original shape due to relaxation of internal stresses, resulting from the previous compression.^27,28^ Therefore, densification on its own does not significantly reduce hydrophilicity and requires proper treatment to reach adequate dimensional stability. This suggests that the effect of densification on chemical resistance against liquid agents is also limited. However, to the authors knowledge, the exact influence on the chemical resistance is not known.

### Motivation

This study explores the concept that modifying the surface layer of laminated veneer lumber (LVL) or plywood with sustainable materials can offer effective protection against harsh substances. So far, most studies have focused on the modification of solid wood, whereas in the current study the modification was carried out at the veneer level. This targeted alteration can optimize material usage, leading to cost efficiency without compromising chemical resistance, drawing on the observation that only the outer layer of wooden structures (beams, panels, etc.) in industrial settings typically experiences chemical erosion, leaving the core intact.^7^ As far as we are aware of the literature, this is the first time that such a detailed, comparative analysis is performed on three different modification methods (acetylation, furfurylation and densification), as a function of surface functionalization.

These wood modification methods are exposed to a wide range of harsh substances, from strong acids to aggressive solvents and strong bases. The investigation is also motivated by the potential application of wood in the field of mechanical engineering and vehicle construction, where the components may be exposed to a wide range of reagents, be it in operation, maintenance or cleaning. The choice of reagents is originally inspired by Ashby’s ‘environmental attack chart’ describing the resistance of materials to six environmental influences, namely strong acids, strong alkalis, solvents, UV radiation, salt water and aerated water.^29^ In contrast to the previous study^30^, in which specific operating and cleaning agents were used as reagents, a generic, more generally applicable approach was deliberately chosen here.

These liquid reagents were selected to test the resistance of wood veneers due to their distinct chemical properties and their potential to affect the wood in various ways. An ionic liquid was included for its ability to potentially dissolve the wood, providing insight into maximum resistance thresholds. Hydrochloric and sulfuric acid were chosen to evaluate the veneers' resistance to acid-induced depolymerization and modification, while sodium hydroxide was used to observe alkaline-induced depolymerization and swelling effects. DMSO was selected for its capacity to partially dissolve and swell the wood, simulating the impact of organic solvents. Lastly, water was used to assess the veneers’ response to simple swelling, a common type of exposure in everyday conditions. The main indicator to assess the efficiency of the protective layer is its tensile strength.

## Materials and Method

Peeled birch wood veneers were supplied by Weitzer Woodsolutions (Weiz Austria), from which a random selection was sent to established industry leaders for modification. The modifications were done by Accsys technologies (Arnhem Netherlands, Accoya, Acetylation), Kebony (Skien, Norway, Kebony, Furfurylation) and Swiss Wood Solutions AG, (Altdorf, Switzerland, Swisswoodsolutions, Densification). In addition to that, some unmodified veneers were kept for use as a reference. The applied wood modification methods are briefly described below.

### Modifications

The acetylation was carried out by Accsys technologies, Arnhem in The Netherlands, using highly concentrated (>90%) acetic anhydride without any catalyst. Achieving a minimum of 19% bound acetyl through a vacuum-pressure impregnation process and subsequent acetylation process at over 120 °C. Kebony, in Skien Norway, performed the furfurylation, using maleic anhydride as a catalyst and a vacuum-pressure process during impregnation. Achieving a weight gain percentage (WGP) of 40% after about 50 h of drying and curing.

### Manufacturing

**Figure 1:**
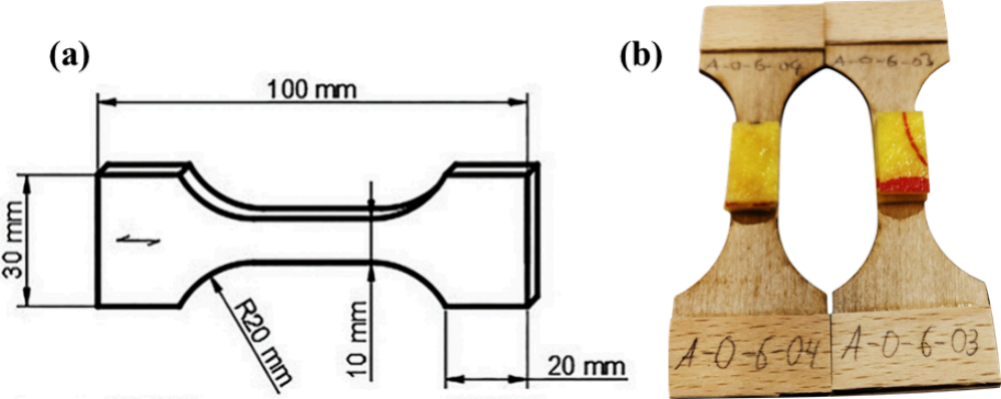
(a) Specimen geometry with 0° grain direction based on Pramreiter et al.^31^ and (b) exemplary specimens with sponge cloth-strips limiting spread of the applied liquids mostly to the central area of the specimen. (Photograph courtesy of Sebastian Wurm. Copyright 2024.).

All veneers have a thickness of 1.5 mm, with the exception of the densified specimen, which exhibit a thickness of 0.8 mm. Dog-bone shaped specimens^31^ were cut from these veneers using a laser-cutter (Trotec Speedy 360 flexx), as seen in Figure 1. In order to prevent failure in the clamping zone, 20 × 30 mm beech wood strips were glued at a 90° angle to the ends of the specimens. A Collano wood glue (RP 3007) was used for this. A total of 560 samples (20 repetitions per variant) were produced and subsequently stored in standard climate conditions (ISO 554, 20 °C, 65% RH)^32^ until equilibrium moisture content was reached.

The four veneer types were subjected to a 24-h treatment involving six chemical reagents. One molar (1 M) Hydrochloric acid (HCl) was employed to assess the veneers' resistance to strong acids, while sulfuric acid (H_2_SO_4_), at the same concentration provided a comparative analysis against another potent acid. Research showed that only strong acids have a significant effect on wood. Such strong acids may break down cellulose and hemicellulose, as well as lignin after prolonged exposure. Additionally, some extractives, such as tannins or resins, may react with the acid. Changing both appearance and stability of the cellular structure.^7,33^ Distilled water served as a neutral control and to observe any effects from pure water exposure, such as swelling. Dimethyl sulfoxide (DMSO) was included in the experiment due to its strong solvent properties, which can break down organic materials. This allowed for the testing of the veneers' resistance to such solvents. 1-ethyl-3-methylimidazolium acetate (Emim-AC), an ionic liquid, was selected to explore the veneer’s stability in the presence of these modern solvents.

These solvents are increasingly used in various industrial applications and able to completely dissolve and delignify wood.^34^ Finally, sodium hydroxide (NaOH) at a concentration of 1 M was employed to assess the durability of the veneers when exposed to strong bases, which may have similar effects as strong acids. Additionally, strong bases may lead to saponification and neutralization of acidic components of the wooden structure.^35^

The 1 M solution of HCl was purchased from Merck KGaA, while the 1 M solution of H_2_SO_4_ was prepared using sulfuric acid (≥95%) obtained from Fischer Scientific. The NaOH solution was prepared using NaOH pastilles (99.2%) sourced from VWR Chemicals. Commercial DMSO (≥99%) was purchased from Sigma-Aldrich, and Emim-AC was supplied by proionic GmbH. One gram of liquid was applied to each specimen. For the contact angle measurements, deionized water and diiodomethane (99%) purchased from Fischer Scientific were used.

### Contact Angle Determination

The contact angles of the unmodified and modified veneers were measured using a Dataphysics OCA200 instrument (Dataphysics, Filderstadt, Germany). This was done before the chemical treatment. The pendant drop technique was utilized with a 1.52 mm diameter tip (Dataphysics, Filderstadt, Germany) attached to a medical syringe. An automated liquid dosing unit was used for dispensing the liquids. Drop sizes were measured in air under climatized laboratory conditions (ISO 187, 23 °C, 50% RH),^36^ and determined using an ellipse fitting algorithm. Drop sizes were maintained at 1 μL for diiodomethane and 2 μL for deionized water. A minimum of 10 measurements per veneer type were taken and averaged.

### FTIR-ATR Spectroscopy

Attenuated total reflection (ATR) measurements were performed on the chemically treated veneers, utilizing a Bruker Alpha FT-IR spectrometer (Billerica, MA, USA), which featured an ALPHA Platinum ATR single reflection diamond module. Spectral scans were conducted across a range of 4000 to 400 cm^–1^, with each measurement averaging 64 scans at a resolution of 4 cm^–1^. Subsequent data analysis was carried out using OriginPro 2020 software.

### Infinite Focus Microscopy

The veneer surfaces, which were in contact with the chemical reagents, were examined using focus variation microscopy with an Alicona InfiniteFocus microscope (model ALC13), with a vertical resolution of 200 nm. For this analysis, 1 × 1 mm images of each sample were captured at 50× magnification. Imaging was conducted using identical light exposure settings for all samples, ensuring that any observed color changes within a specific veneer type can be attributed solely to the effects of the chemical treatment.

### Experimental Determination of Chemical Resistance

The specimens were prepared for application of the chemical reagents by placing a number of them inside a glass container and positioning a stack of three sponge cloth strips (20 × 10 mm) at the center of each specimen (Figure 1b).

After preparing the samples as described, one gram of the respective test liquid was applied to each sample. Every sample was removed from its container after exactly 24 h of exposure at standard climate conditions (ISO 554, 20 °C, 65% RH).^32^ The Sponge cloth-strips as well as any excess liquid were removed using tweezers prior to photographing and weighing. The time between removal of the sample and starting the test had to be kept to a minimum, in order to prevent further degradation of the wood veneer. Around one to 2 min passed before starting the tensile test using a Zwick-Roell universal testing machine, at a speed of 2 mm/min and a force cutoff threshold of 50%.

After testing, each specimen was photographed again and then placed in a water bath in order to wash out all remaining test liquid. This was done to prevent further degradation and remove any residual chemicals, which could interfere with subsequent tests. A separate container was used for each chemical reagent and the water was then changed daily for a period of 13 days. FTIR-ATR spectroscopy and IFM were done with cleaned and dried samples.

### Statistical Analysis Techniques

ANOVA, Levene’s test, the Shapiro-Wilk test and the Kruskal–Wallis test were conducted on the results for each modification type. The ANOVA requires independency, homogeneity of variances and normality within each group. The Levene’s test was performed to demonstrate homogeneity of variances and the Shapiro-Wilk test was used to prove normality of the test data. In addition to the ANOVA, the Kruskal–Wallis one-way analysis of variance was conducted, which makes no assumption on normality and is less sensitive to outliers. Furthermore, the unpaired *t* test was performed in order to find out which particular chemical reagent had a significant influence on the tensile strength. This test is used to check the similarity of two groups and gives a t-value, which is then compared to a *t*-critical. In this case, the condition −*t*_crit_ < *t* < *t*_crit_ must be satisfied to prove that no statistically significant difference between the two groups exists.

## Results and Discussion

### Penetration Behavior

Most liquids for this study exhibit low viscosity and were observed to spread into the clamping zone, as well as spill over the edge of the central area. Both cases are detrimental for the determination of liquid penetration through observation of darkening of the back side. So the first challenge was to prevent the spread of the testing liquids on the specimen. Blotting paper was used in earlier research to hold the applied liquid and act as a reservoir^30^ but was found to be inadequate against strong acids and bases. Prior to the experiments, a number of different sponge materials were subjected to preliminary testing. The final choice fell on “Vileda” general-purpose sponge cloth (Figure 1b), due to its ability to quickly absorb and steadily release liquid, as well as withstand the various aggressive chemicals over more than 24 h.

**Figure 2:**
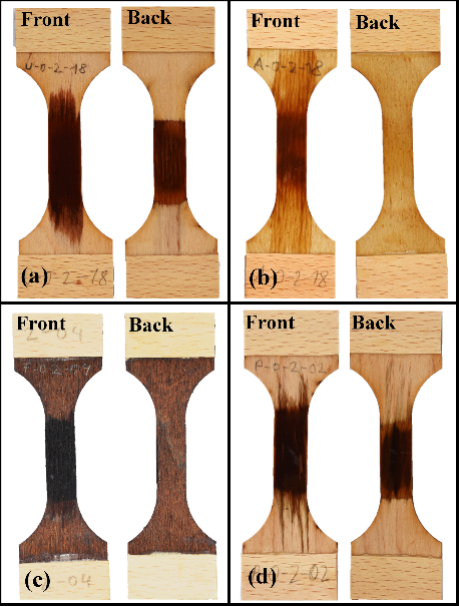
Photographs of (a) unmodified, (b) acetylated, (c) furfurylated and (d) densified veneers after 24 h of exposure to NaOH. (Photographs courtesy of Sebastian Wurm. Copyright 2024).

Twenty-four hours of exposure to the testing liquids led to complete penetration of the unmodified and densified veneers, as shown by the darkening of the backside, seen in Figure 2a,d. No such darkening was noticeable on the backside of the acetylated and furfurylated specimens (Figure 2b,c). This suggests that in a laminated wood structure with acetylated or furfurylated veneers as the outer layers and unmodified wood underneath, even 1.5 mm thin acetylated or furfurylated veneers are capable of preventing liquid penetration into the underlying unmodified wood.

### IFM Analysis

**Figure 3:**
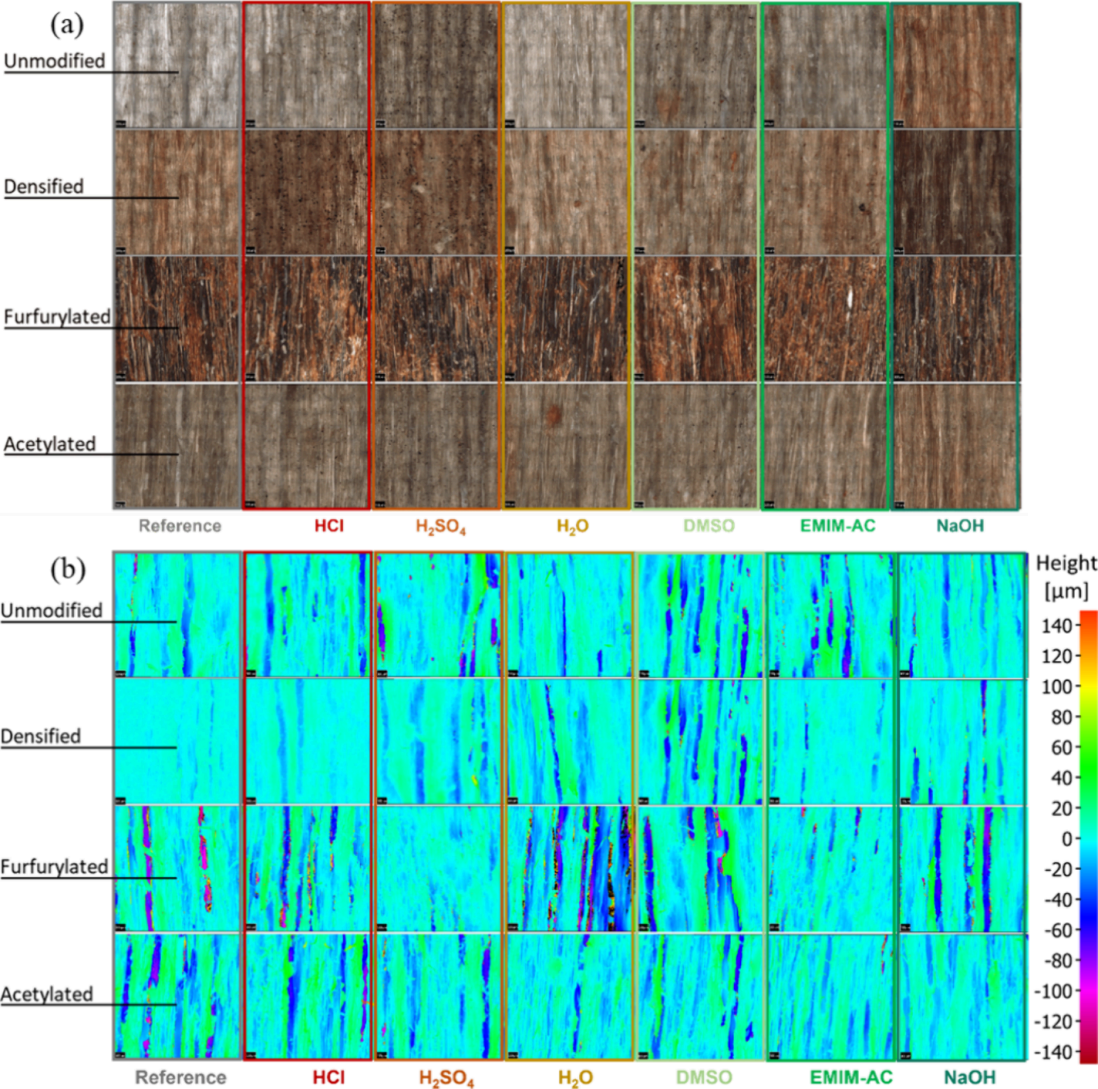
(a) IFM images of different wood veneers, illustrating both chemically treated and untreated (reference) samples, (b) Topological IFM images of different wood veneers, illustrating both chemically treated and untreated (reference) samples.

The IFM images (Figure 3a) revealed that the exposure to 1 M HCl and H_2_SO_4_ resulted in the formation of dark spots on the surface of all wood veneers, with the exception of the furfurylated samples. The inherent dark coloring of the furfurylated veneers likely masked the visibility of these spots. In contrast, treatment with DMSO induced spot formation only on the reference and acetylated veneers. All samples exhibited uniform discoloration following exposure to 1 M NaOH. The discoloration of unmodified and densified veneers was also evident after the dilute acid treatment with HCl and H_2_SO_4_.

The color changes of wood can be attributed to chemical interactions and structural changes within the wood components. Hydrolysis can cause water-soluble extractives such as phenolic compounds to migrate to the surface and undergo oxidation, resulting in colored substances. Other contributing factors include the degradation and subsequent condensation of lignin. Lignin degradation produces free radicals, which react with oxygen to form chromophoric groups. These chromophores could be unsaturated carbonyl compounds such as quinones and/or ketones.^37^ From the analysis of these IFM images, it is not possible to determine the specific structural changes that lead to the formation of dark spots versus those that cause uniform discoloration. However, it is evident that while furfurylation and acetylation treatments prevent uniform discoloration, they do not inhibit the formation of dark spots.

The topological view of the IFM images (Figure 3b) reveals variations in surface roughness both between different veneer types and within the same type when treated with different chemicals. Among the reference samples, the densified veneer exhibits the smoothest surface, whereas the furfurylated veneer displays the greatest roughness. Chemical treatments alter the roughness of densified veneers, as the reagents induce swelling that is most apparent in this type. However, topological differences within a veneer type cannot be solely attributed to the treatment with different chemicals. The inherent heterogeneity of the veneer surfaces complicates the ability to draw definitive conclusions about the entire surface structure from 1 × 1 mm IFM image sections

### Mass Increase

**Figure 4:**
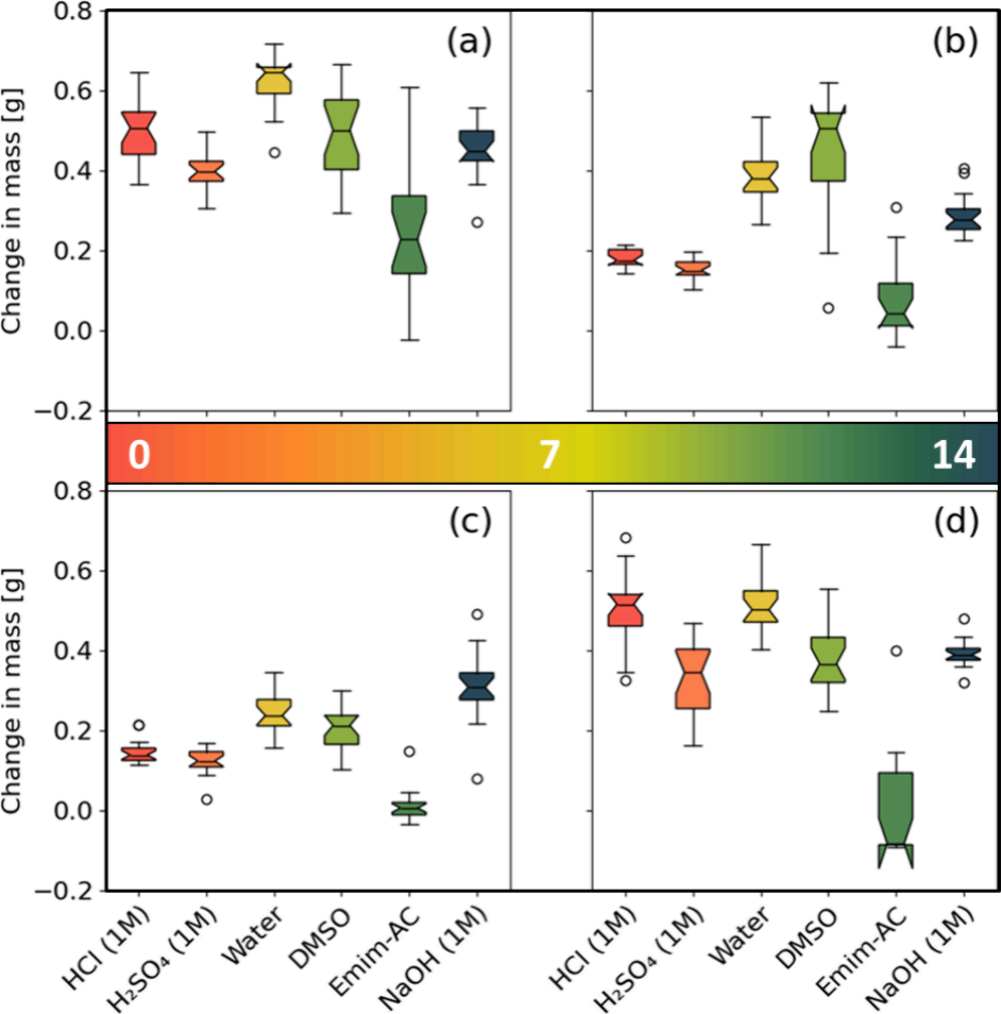
Absolute change in mass of (a) unmodified, (b) acetylated, (c) furfurylated and (d) densified veneers brought about by each chemical reagent, colored according to the pH of their respective chemical reagent.

The largest overall increase in mass (Figure 4) was caused by distilled water with an average increase of 0.43 g, closely followed by DMSO and NaOH with 0.38 and 0.37 g, respectively. The lowest overall absorption was observed with the ionic liquid Emim-AC with an average increase of 0.07 g. Densification in general, has a limited effect in preventing liquid absorption. Emim-AC appears to be the only exception as it hardly penetrates the veneer. This is also true for the other two modification types, but they also drastically reduce the absorption of all other test liquids. The furfurylated specimens did especially well in this regard, as they reduced the mass increase of DMSO and water in particular to a mere 0.20 and 0.24 g down from 0.48 and 0.62 g, respectively. In short, densification decreases the average liquid absorption by about 29%, while acetylation and furfurylation achieve around 46 and 73% reduction, respectively.

### Tensile Strength

**Figure 5:**
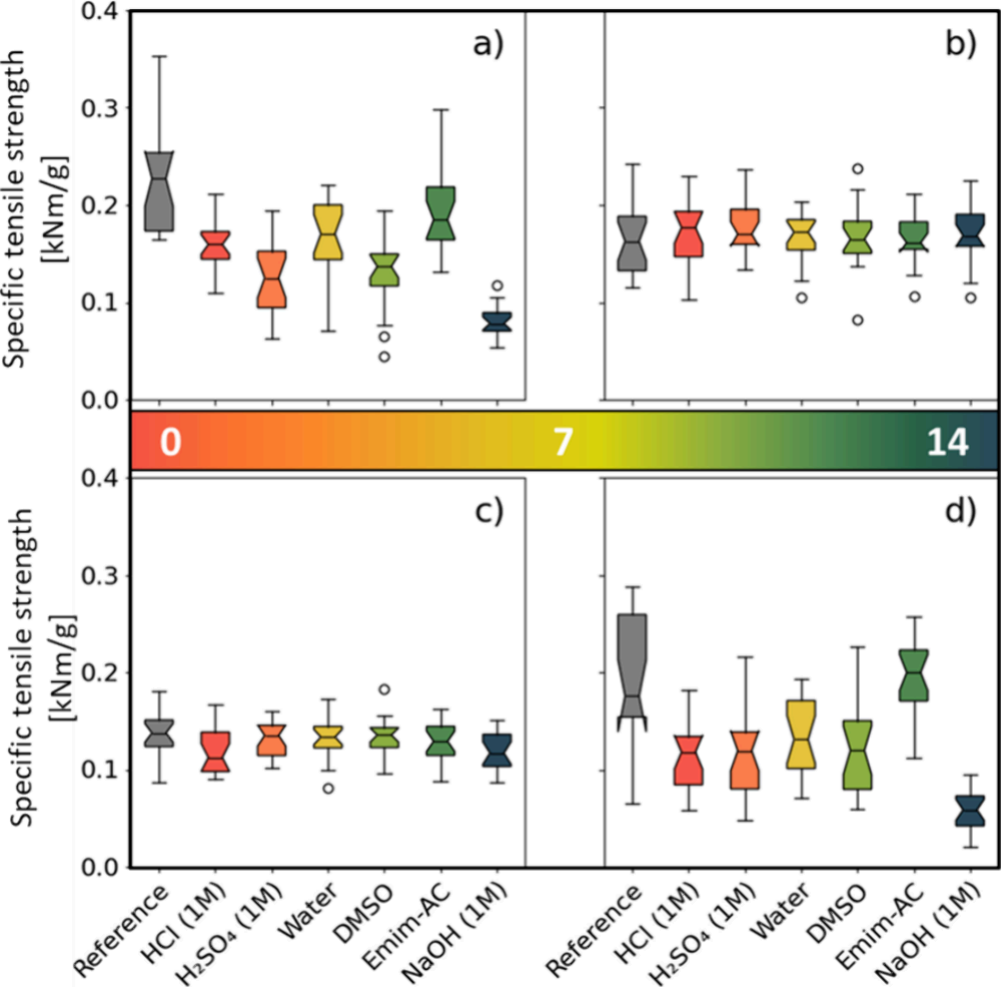
Specific tensile strength of (a) unmodified, (b) acetylated, (c) furfurylated and (d) densified veneers for each test group, colored according to the pH-value of their respective chemical reagent.

Unmodified and densified veneers appear to be significantly affected by chemical reagents, while acetylated and furfurylated veneers show greater consistency with the reference samples (Figure 5). The specific tensile strength was calculated using the original cross sectional area and density of the specimens (conditioned at 20 °C and 65% RH), in order to ensure comparability of the results. It is used as a measurement of the combined effect of the degradation of the material over 24 h and the swollen state of the veneer due to liquid absorption.

The Shapiro-Wilk test confirms normality of each test group within the four populations. The results of the ANOVA suggest that there is no statistically significant difference in the mean specific tensile strength of the acetylated samples (*p* = 0.87 > 0.05). Furthermore, the Kruskal–Wallis test leads to believe that acetylated (*p* = 0.85 > 0.05) as well as the furfurylated (*p* = 0.051 > 0.05) specimens show no statistically significant difference. However, ANOVA and the Kruskal–Wallis test agree when it comes to the unmodified (*p* = 2.74 × 10^–22^ < 0.05 and *p* = 2.54 × 10^–15^ < 0.05) and densified veneers (*p* = 5.61 × 10^–20^ < 0.05 and *p* = 3.09 × 10^–14^ < 0.05). Both tests show clearly that there is a statistically significant difference in specific tensile strength for these two kinds of modifications.

The results of the unpaired *t* test show that every chemical reagent has a significant influence on the unmodified veneer. Physical densification seems to only provide resistance against Emim-AC. Furfurylation protects against every reagent except sodium hydroxide and hydrochloric acid, whereas acetylation seems to provide full resistance against every chemical reagent used based on the results of the unpaired *t* test.

The modification types may be grouped in two clusters. On the one hand unmodified and densified veneers form the first clusters and on the other hand, acetylated and furfurylated veneers form the second clusters.

Regarding the results of the first cluster, they indicate that sodium hydroxide has the largest impact on the mechanical properties, resulting in an average of 70 and 64% reduction in specific tensile strength of the densified and unmodified veneers, respectively. Sulfuric acid and DMSO may be grouped together, as their impact on the mechanical properties are quite similar. These chemicals result in an average reduction of 44 and 43% on the unmodified reference specimens, respectively. Regarding the densified specimens, the average reductions amount to 40 and 42% respectively. Next, hydrochloric acid and distilled water seem to have a lower influence on unmodified veneers (30 and 27% reduction) than on densified veneers (42 and 32% reduction). The lowest influence seems to result from the exposure to Emim-AC, amounting to an average reduction ranging from 15% to almost 0%. This could be due to the high viscosity of Emim-AC at room temperature, resulting in low penetration of the wood’s surface or because of its lower pH value of around 11 compared to 14 of the one molar NaOH solution.

The results of the second cluster, including acetylated and furfurylated specimens, show that only sodium hydroxide and hydrochloric acid seem to have any statistically significant influence on the mechanical properties. Amounting to a 13% reduction in specific tensile strength for the furfurylated veneers. Whereas no significant influence of any chemical reagent could be observed on the acetylated veneers.

### FTIR-ATR Spectroscopy Analysis

**Figure 6:**
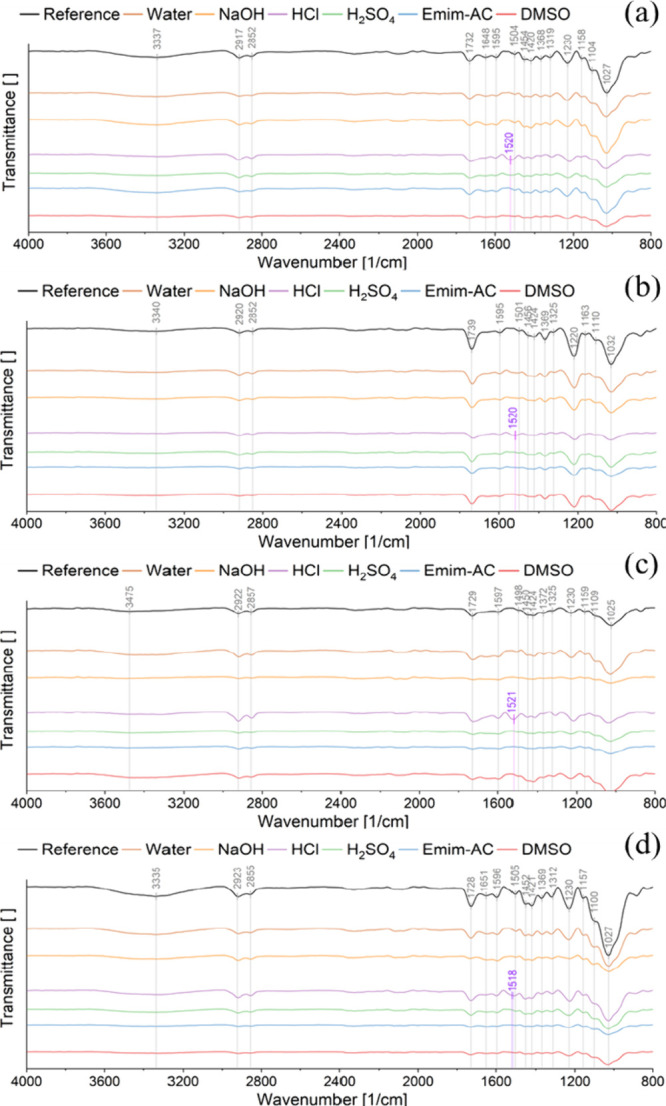
ATR-IR spectra of (a) unmodified, (b) acetylated, (c) furfurylated and (d) densified veneers subjected to different chemicals, as well as chemically untreated reference samples (indicated as reference in the plots).

While IFM gave some hints on specific reactions taking place in the wood samples, IR spectroscopy is capable of revealing more information on the presence of functional groups. In our ATR-IR measurements, the exact wavenumbers of specific bands varied slightly among different specimens. To enhance clarity, we report rounded wavenumbers. The exact wavenumbers of the bands can be seen in Figure 6.

The low band intensities observed in some specimens can be attributed to poor contact of the ATR crystal with the veneers, primarily because of the high surface roughness (Figure 6b). For example, this may explain the lack of a prominent OH stretching band in some chemically treated samples. Certain treatments cause swelling, increase surface roughness, and thereby diminish overall signal intensity, potentially masking the broad OH band. In contrast, the densified reference sample, with its reduced surface roughness, achieves much better contact with the ATR crystal, resulting in the strongest signal. These variations in contact lead to inconsistencies in band intensities, which is why our analysis focuses primarily on qualitative aspects for the chemically treated samples. Nonetheless, certain notable differences in band intensities between the reference samples are evident and should be further discussed. For instance, when comparing the unmodified reference veneers with the acetylated ones, the OH stretching intensity significantly decreases due to acetylation, indicating a reduction in the amount of OH groups. The intensity of the band at 1730 cm^–1^, associated with acetyl groups present in hemicelluloses, increased with acetylation and shifted slightly to 1739 cm^–1^. Similarly, the intensities of the bands at 1370 cm^–1^ (cellulose and hemicellulose) and 1230 cm^–1^ (lignin and hemicellulose) also increased as a result of the acetylation. This indicates that the main wood components, hemicellulose, cellulose, and lignin have undergone acetylation. As the 1739, 1370, and 1230 cm^–1^ bands do not disappear when the acetylated veneers are in contact with chemical reagents, it can be assumed that the acetylated samples are chemically resistant. The IR spectrum of the reference sample of the furfurylated veneers also displays a notable reduction in the OH stretching intensity compared to unmodified and densified veneers. Moreover, the band at 1650 cm^–1^, associated with the conjugated carbonyl (C=O) stretching in lignin, is absent in the reference samples of both acetylated and furfurylated veneers. This absence suggests that the chemical modifications not only target the hydroxyl groups but also disrupt the original conjugated system of the lignin.^38^

Across all chemical reagents, the treatment with 1 M HCl had the most severe effects on the IR spectra of the veneers. A band at around 1520 cm^–1^ appears, which can be attributed to modifications in the aromatic lignin structure.^39^ The band at 1730 cm^–1^ broadened, indicating the emergence of new overlapping bands between 1700 and 1730 cm^–1^. It has been reported that during treatment with dilute acids, cellulose and hemicellulose are hydrolyzed into glucose and xylose, which subsequently degrade into compounds such as furfural (FF) and 5-hydroxymethylfurfural (HMF). Reaction intermediates are then generated from FF and HMF, which can react with FF and HMF to form an acid-insoluble material known as pseudolignin. Pseudolignin, which is rich in carbonyl, carboxyl, aromatic, methoxy, and aliphatic structures is characterized by strong bands around 1700 cm^–1^ attributed to C=O stretching in carboxylic acids, aromatic esters or aliphatic ketones.^40−43^ Therefore, it can be inferred that the treatment with 1 M HCl resulted in the formation of pseudolignin on all veneers. This phenomenon was not observed when treating modified veneers with 1 M H_2_SO_4_; only the unmodified veneer displayed the band around 1520 cm^–1^ and the broadening of the 1730 cm^–1^ band. It remains unclear whether these modifications inhibited pseudolignin formation or if the absence of these bands was caused by poor signal quality resulting from insufficient contact with the ATR crystal. In order to precisely confirm it as pseudolignin, further analyses, such as solid-state NMR, would be required. The formation of pseudolignin is linked to the degradation of lignin into carbonyl compounds, which contribute to color changes in the wood. These lignin degradation products could explain the observed spot formation and discoloration in the acid-treated veneers.

### Wettability of the Wood Veneers

**Figure 7:**
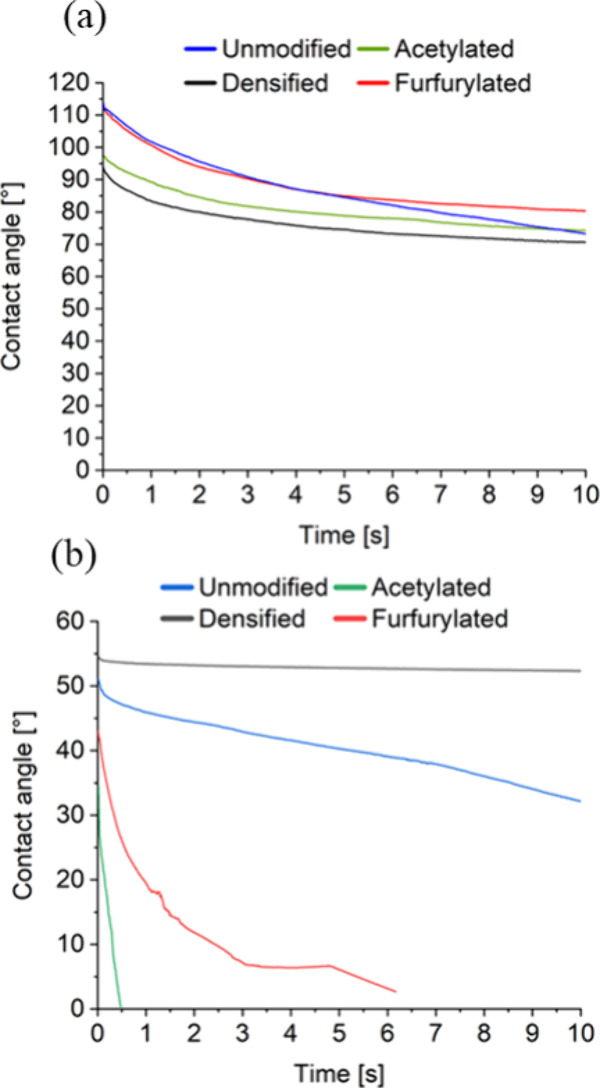
Contact angle measurements of the reference samples of modified wood veneers with (a) deionized water and (b) diiodomethane.

Figure 7 depicts the contact angles of the reference samples of modified wood veneers with deionized water (a) and diiodomethane (b). The contact angle of water on the unmodified sample decreases within 10 s, unlike the nearly static contact angles observed on modified samples. Therefore, it can be assumed that the modifications of the veneers lowered the interaction strength with water. The measurement of the contact angle with diiodomethane reveals substantial differences in wetting duration between chemically modified and the densified and unmodified veneers. For furfurylated and acetylated veneers, the contact angle decreases to below 10° in less than 10 s. This suggests that the chemical modifications increased the veneers’ nonpolar surface components, enhancing hydrophobicity and resistance to water exposure. The FTIR analysis of the furfurylated and acetylated reference samples supports this by showing a reduction or absence of OH groups in contrast to unmodified and densified reference samples, which are key contributors to hydrophilicity. With fewer OH groups, the veneers interact more with nonpolar liquids like diiodomethane and less with water, confirming improved water resistance of the chemically modified veneers. This suggests that the reduction of free OH-groups through acetylation and furfurylation enhances interaction with a less polar liquid like diiodomethane. The decreased wettability correlates with improved tensile strength retention, particularly after exposure to harsh chemicals like NaOH.

The contact angles with deionized water and diiodomethane observed on the veneers is significantly influenced by surface roughness, making it relatively unreliable to directly deduce hydrophilicity from the contact angle values alone. However, the wetting times of the droplets on the samples can be used to infer the interaction strength between the surface and the test liquid, providing a comparative measure of the samples. Thus, the combined insights from CA measurements and FTIR analysis provide a thorough understanding of the surface modifications’ effects, confirming the improved water resistance of the treated veneers.

## Conclusions and Outlook

While the chemical resistance of the birch veneers was massively improved by either acetylation or furfurylation, the same cannot be said for the densification, which resulted in almost identical resistance to the unmodified reference samples. In the case of the acetylated and furfurylated samples, there was almost no significant reduction in tensile strength, regardless of the chemicals applied. This can be explained by the increased hydrophobicity due to nonpolar surface components. In addition, FTIR analysis shows a reduction in the available OH groups, contributing to a reduced ability to absorb liquids. Interestingly, esters, which are generally unstable in solution when exposed to strong acids and bases, demonstrated remarkable resilience in veneers. The limited accessibility and altered kinetics in this configuration resulted in minimal observable cleavage of ester bonds. Consequently, the mechanical properties, particularly tensile strength, were largely preserved. The spectra indicated minimal changes in vibrational modes associated with the ester functional groups, further substantiating the lack of significant bond cleavage. While there was a noticeable change in sample coloration after acid exposure, indicating some degree of modification, this alteration does not adversely affect the mechanical characteristics.

In contrast, the densified and unmodified veneers were severely affected by most of the chemicals, resulting in a deterioration of their capabilities under tensile loading. Therefore, acetylation and furfurylation is a promising strategy for achieving chemical resistance in load-bearing wooden components. This is particularly relevant in construction industry and in mechanical engineering where the wood needs to be protected against environmental impacts. This study was carried out at veneer level to determine the potential suitability of modified veneers as a protective outer layer of wooden beams. This would enable a more sustainable and economical design of chemically resistant wooden load bearing elements, by limiting the modification to a fraction of the beam volume. In addition, the use of modified wood veneers arguably increases their lifetime, thereby prolonging the CO2 storage effect in the materials, contributing to a better ecologic footprint.

## Supporting Information Available

Initial densities of all specimen with statistical analyses (ANOVA, Levene’s test, Kruskal–Wallis test, Shapiro-Wilk test, unpaired t-test); changes in specific tensile strength with statistical analyses (ANOVA, Levene’s test, Kruskal–Wallis test, Shapiro-Wilk test, unpaired t-test); and assignment of IR bands typical for wood samples (PDF)
