# Supplementary material for: Chemical Resistance of Modified Wood Veneers in Sustainable Load Bearing Elements
Source: ACS Omega. 2024 Nov 20;9(48):47690–8. doi: 10.1021/acsomega.4c07320 (PMC11618423; doi:10.1021/acsomega.4c07320)
Supplement: Supplementary file 1 — ao4c07320_si_001.pdf [file ao4c07320_si_001.pdf]

Supplementary Information

Chemical resistance of modified wood veneers in sustainable load bearing elements

Sebastian Wurm[1], Alexa Scheer[2], Georg Baumann[1], Markus Wagner[1], Kevin Vitzthum[3], Stefan Spirk[2], Florian Feist[1]**

1 Vehicle Safety Institute, Graz University of Technology, Inffeldgasse 13/6, 8010 Graz, Austria

2 Institute of Bioproducts and Paper Technology, Graz University of Technology, Inffeldgasse 23, 8010 Graz, Austria

3 W.E.I.Z. Forschungs & Entwicklungs GmbH, Franz-Pichler-Straße 30, 8160 Weiz, Austria

Corresponding Authors: florian.feist@tugraz.at, stefan.spirk@tugraz.at

Number of pages: 8

Number of tables: 3

Number of figures: 2

Initial densities

The density of each specimen was determined prior to chemical exposure and is depicted in Figure S1. Each of the four modification types contains seven test groups, one for each chemical reagent plus the untreated reference group. The average densities of unmodified and acetylated are relatively similar with 499.13 ± 13.65 kg/m³ and 505.16 ± 3.39 kg/m³ respectively, whereas the furfurylated samples are of slightly higher average density with 661 ± 7.16 kg/m³. Naturally, the densified samples have the highest average density at 1000 ± 13.88 kg/m³. The difference of densities within each kind of wood modification seem to be low for the acetylated and furfurylated veneers and relatively higher for the unmodified and densified veneers. In order to verify if there actually is a statistically significant difference in initial densities, the analysis of variance (ANOVA) was performed.

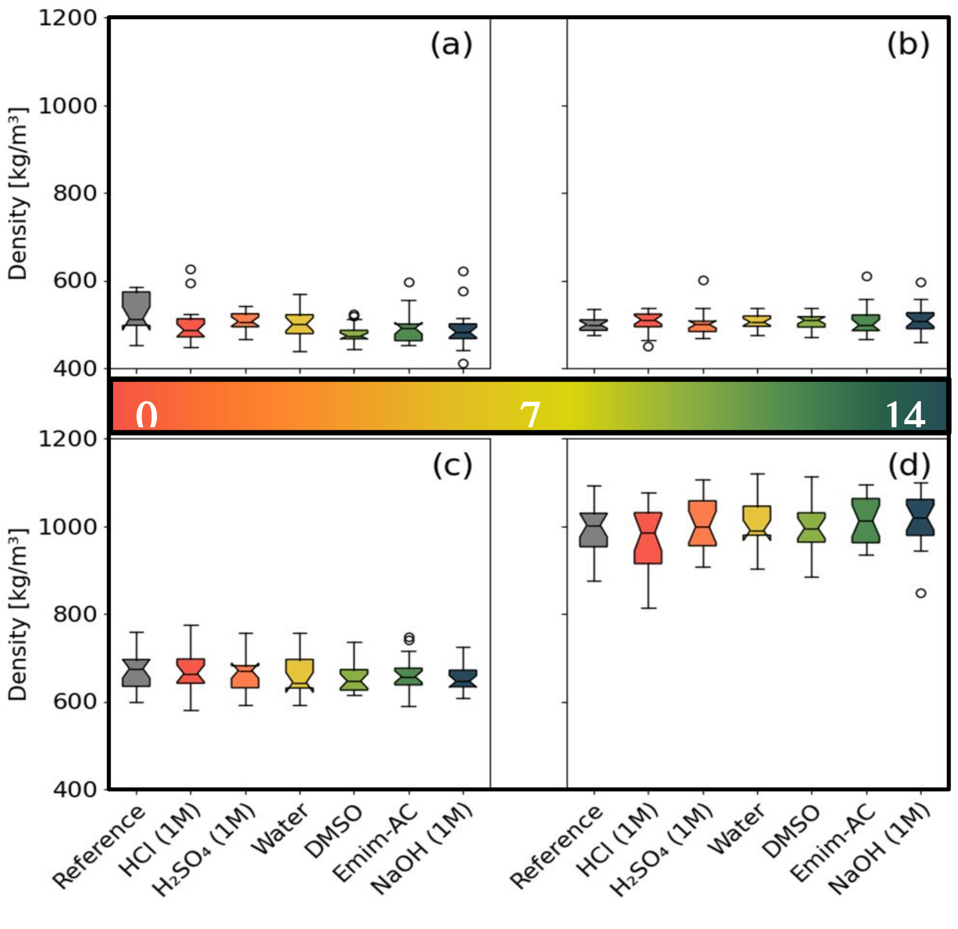

Figure S1. Boxplots of the initial densities (before exposure to chemicals) of unmodified (a), acetylated (b), furfurylated (c) and densified (d) veneers, colored according to the pH of their respective chemical reagent.

For three of the four modification groups, namely the acetylated ($p=0.88>0.05$), furfurylated ($p=0.67>0.05$) and densified ($p=0.29>0.05$), no statistically significant difference in mean density was found and thus no influence on the tensile strength is expected. All three of these groups exhibit Levene's p-values and Shapiro-Wilk p-values greater than 0.05, proving homogeneity of variances and normality. The sole exceptions are the acetylated samples used for treatment with sulfuric acid and Emim-AC, which have Shapiro-Wilk p-values of 0.01 and 0.012 respectively. However, the Kruskal-Wallis test for these groups ($p=0.648>0.05$) agrees with the ANOVA, therefore no statistically significant difference for the initial densities of the acetylated samples can be assumed.

A statistically significant difference between the initial densities is only found for the unmodified veneers, according to the ANOVA as well as the Kruskal-Wallis test. However, the density is not the most influential factor on the tensile strength of wood along the grain according to literature (*1, 2*). Therefore, the results of the unmodified veneers can still be used to evaluate the influence of the chemical treatment on the tensile strength.

Table S1. Results of the Shapiro-Wilk test, the ANOVA, Levene's test and the Kruskal-Wallis test for the initial densities of each sample group, sorted by modification type.

**Unmodified veneer**	Reference	HCl	H_2SO_4	Water	DMSO	Emim-AC	NaOH

Mean density [kg/m³]	525.0	500.1	507.5	501.2	479.0	492.3	488.8
Variance	1556.3	1932.0	432.2	1287.7	510.1	1375.4	2093.5
Shapiro-Wilk p-value	0.03	0.01	0.91	0.93	0.08	0.01	0.02
ANOVA p-value				0.004 < 0.05			
Levene's test p-value				0.135 > 0.05			
Kruskal-Wallis p-value				0.0008 < 0.05			

**Acetylated veneer**	Reference	HCl	H_2SO_4	Water	DMSO	Emim-AC	NaOH
Mean density [kg/m³]	501.3	505.1	500.7	506.4	504.4	506.9	511.5
Variance	342.3	632.1	852.0	284.1	373.7	1199.7	1069.9
Shapiro-Wilk p-value	0.12	0.08	0.01	0.86	0.37	0.01	0.45
ANOVA p-value				0.881 > 0.05			
Levene's test p-value				0.225 > 0.05			
Kruskal-Wallis p-value				0.648 > 0.05			

**Furfurylated veneer**	Reference	HCl	H_2SO_4	Water	DMSO	Emim-AC	NaOH
Mean density [kg/m³]	671.6	669.7	664.6	656.5	651.2	661.2	654.3
Variance	2158.8	2368.5	1901.2	2195.6	964.7	1842.6	1001.8
Shapiro-Wilk p-value	0.45	0.94	0.47	0.35	0.06	0.34	0.44
ANOVA p-value				0.671 > 0.05			
Levene's test p-value				0.329 > 0.05			
Kruskal-Wallis p-value				0.713 > 0.05			

**Densified veneer**	Reference	HCl	H_2SO_4	Water	DMSO	Emim-AC	NaOH
Mean density [kg/m³]	990.8	972.3	1004.9	1003.4	997.5	1014.5	1015.7
Variance	4194.9	5495.6	3943.4	2604.1	2875.2	2761.9	3609.7
Shapiro-Wilk p-value	0.40	0.45	0.39	0.54	0.95	0.15	0.26

ANOVA p-value	0.292 > 0.05
Levene's test p-value	0.356 > 0.05
Kruskal-Wallis p-value	0.476 > 0.05

Figure S2 presents an overview of the change in specific tensile strength resulting from each chemical reagent for all four modification types. Note that, the positive changes on the acetylated specimens do not imply a positive effect of the chemical reagent on the specimen, but rather result from the inherently large variance of the veneers, obscuring the limited effect of the chemicals.

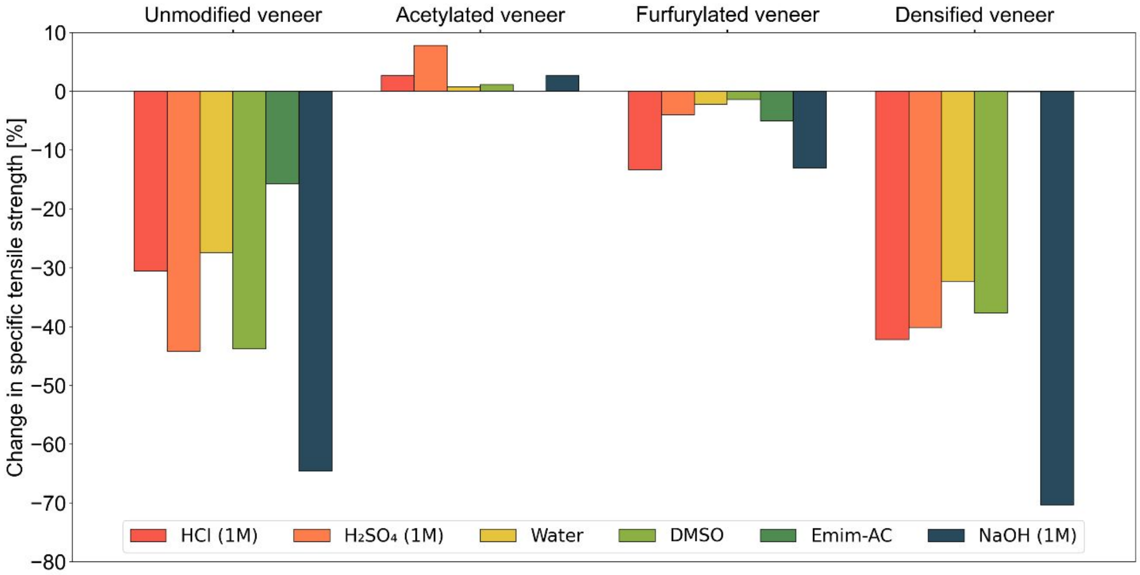

Figure S2. Overview of the average percentage changes in specific tensile strength following the exposure to each chemical reagent, colored according to the pH-value of their respective chemical reagent.

Table S2. Results of the Shapiro-Wilk test, the ANOVA, Levene's test and the Kruskal-Wallis test for the specific tensile strength of each sample group, sorted by modification type.

**Unmodified veneer**	Reference	VS. HCl	VS. H2SO4	VS. Water	VS. DMSO	VS. Emim-AC	VS. NaOH
Mean specific tensile strength [MPa/(kg/m³)]	0.228	0.158	0.127	0.166	0.128	0.192	0.081
Variance	0.003	0.0008	0.0015	0.0019	0.0012	0.0022	0.0003
Shapiro-Wilk p-value	0.09	0.75	0.53	0.10	0.22	0.34	0.84
Unpaired t-Test t-value	-	5.04	6.69	4.00	6.85	2.22	11.53
T-critical two-tail	-	2.05	2.02	2.02	2.04	2.02	2.07
**Significant Influence?**	-	**yes**	**yes**	**yes**	**yes**	**yes**	**yes**
ANOVA p-value				2.735E-22 < 0.05			
Levene's test p-value				0.002 < 0.05			
Kruskal-Wallis p-value				2.544E-15 < 0.05			

**Acetylated veneer**	Reference	VS. HCl	VS. H2SO4	VS. Water	VS. DMSO	VS. Emim-AC	VS. NaOH
Mean specific tensile strength [MPa/(kg/m³)]	0.1646	0.1690	0.1775	0.1659	0.1666	0.1647	0.1690
Variance	0.0012	0.0014	0.0010	0.0007	0.0010	0.0006	0.0010
Shapiro-Wilk p-value	0.4721	0.4767	0.3032	0.3832	0.2880	0.4728	0.5804
Unpaired t-Test t-value	-	-0.38	-1.23	-0.13	-0.18	0.00	-0.41
T-critical two-tail	-	2.02	2.02	2.02	2.02	2.02	2.02
**Significant Influence?**	-	**no**	**no**	**no**	**no**	**no**	**no**
ANOVA p-value				0.869 > 0.05			
Levene's test p-value				0.485 > 0.05			
Kruskal-Wallis p-value				0.853 > 0.05			

**Furfurylated veneer**	Reference	VS. HCl	VS. H2SO4	VS. Water	VS. DMSO	VS. Emim-AC	VS. NaOH

Mean specific tensile strength [MPa/(kg/m³)]	0.1362	0.1181	0.1308	0.1332	0.1343	0.1293	0.1185
Variance	0.0006	0.0005	0.0003	0.0006	0.0004	0.0004	0.0004
Shapiro-Wilk p-value	0.9306	0.0879	0.3028	0.6082	0.8256	0.4454	0.3942
Unpaired t-Test t-value	-	2.38	0.79	0.38	0.27	0.98	2.43
T-critical two-tail	-	2.02	2.02	2.02	2.02	2.02	2.02
**Significant Influence?**	-	**yes**	**no**	**no**	**no**	**no**	**yes**
ANOVA p-value	0.03 < 0.05						
Levene's test p-value	0.774 > 0.05						
Kruskal-Wallis p-value	0.051 > 0.05						

**Densified veneer**	Reference	VS. HCl	VS. H2SO4	VS. Water	VS. DMSO	VS. Emim-AC	VS. NaOH
Mean specific tensile strength [MPa/(kg/m³)]	0.1938	0.1120	0.1160	0.1312	0.1207	0.1937	0.0575
Variance	0.0038	0.0014	0.0018	0.0017	0.0021	0.0017	0.0004
Shapiro-Wilk p-value	0.1324	0.3711	0.7419	0.1549	0.3774	0.7827	0.9662
Unpaired t-Test t-value	-	5.0739	4.6676	3.7786	4.2619	0.0049	9.4059
T-critical two-tail	-	2.0395	2.0244	2.0345	2.0244	2.0345	2.0687
**Significant Influence?**	-	**yes**	**yes**	**yes**	**yes**	**no**	**yes**
ANOVA p-value	5.613E-20 < 0.05						
Levene's test p-value	0.0006 < 0.05						
Kruskal-Wallis p-value	3.094E-14 < 0.05						

Table S3. Assignment of IR bands typical for wood samples.

**Wavenumber [cm⁻¹]**	**Assignment**
1030	C-O stretching in cellulose and hemicelluloses

1110	Asymmetric C-O-C stretching in cellulose and hemicelluloses
1160	Asymmetric C-O and C-C stretching
1230	Syringyl ring and C-O stretching in lignin and hemicellulose
1320	C-H and C-O vibration in the syringyl ring of cellulose and lignin
1370	C-H deformation in cellulose and hemicelluloses
1420	Vibrations of the aromatic lignin structure
1450	C-H deformation in lignin and carbohydrates
1500	Aromatic C=C vibrations of lignin
1595	Aromatic C=C vibrations of lignin
1650	Conjugated C=O stretching in lignin
1730	Un-conjugated C=O stretching in hemicellulose
2855	Symmetric C-H vibrations related to methyl and methylene groups
2920	Asymmetric C-H vibrations of methyl and methylene groups
3300-3500	O-H stretching vibrations in cellulose and hemicellulose

References

1. Ehrhart, T.; Steiger, R.; Palma, P.; Frangi, A. *Estimation of the tensile strength of European beech timber boards based on density, dynamic modulus of elasticity and local fibre orienation.*

2. Pramreiter, M.; Stadlmann, A.; Huber, C.; Konnerth, J.; Halbauer, P.; Baumann, G.; Müller, U. The Influence of Thickness on the Tensile Strength of Finnish Birch Veneers under Varying Load Angles. *Forests* **2021,** *12* (1), 87. DOI: 10.3390/f12010087.
